# Crystal Structure, Solubility, and Pharmacokinetic Study on a Hesperetin Cocrystal with Piperine as Coformer

**DOI:** 10.3390/pharmaceutics14010094

**Published:** 2022-01-01

**Authors:** Yanjie Liu, Fan Yang, Xiuhua Zhao, Siying Wang, Qilei Yang, Xiaoxue Zhang

**Affiliations:** 1 College of Chemistry, Chemical Engineering and Resource Utilization, Northeast Forestry University, Harbin 150040, China; klp15lyj@nefu.edu.cn (Y.L.); double_g123@nefu.edu.cn (F.Y.); wsy0822@nefu.edu.cn (S.W.); yql@nefu.edu.cn (Q.Y.); zhangxiaoxue@nefu.edu.cn (X.Z.); 2 Key Laboratory of Forest Plant Ecology, Ministry of Education, Northeast Forestry University, Harbin 150040, China; 3 Heilongjiang Provincial Key Laboratory of Ecological Utilization of Forestry-Based Active Substances, Northeast Forestry University, Harbin 150040, China

**Keywords:** hesperetin, piperine, cocrystal, solubility, bioavailability

## Abstract

Hesperetin (HES) is a key biological active ingredient in citrus peels, and is one of the natural flavonoids that attract the attention of researchers due to its numerous therapeutic bioactivities that have been identified in vitro. As a bioenhancer, piperine (PIP) can effectively improve the absorption of insoluble drugs in vivo. In the present study, a cocrystal of HES and PIP was successfully obtained through solution crystallization. The single-crystal structure was illustrated and comprehensive characterization of the cocrystal was conducted. The cocrystal was formed by two drug molecules at a molar ratio of 1:1, which contained O–H–O hydrogen bonds between the carbonyl and ether oxygen of PIP and the phenolic hydroxyl group of HES. In addition, a solubility experiment was performed on powder cocrystal in simulated gastrointestinal fluid, and the result revealed that the cocrystal improves the dissolution behavior of HES compared with that of the pure substance. Furthermore, HES’s bioavailability in the cocrystal was six times higher than that of pristine drugs. These results may provide an efficient oral formulation for HES.

## 1. Introduction

Numerous drugs are widely known to have polymorphism which leads to differences in bioavailability and activity indicators. Statistics show that approximately 85% of marketed drugs are crystal products, and most clinical drugs have structural specificity. Therefore, a drug’s crystal structure is a key factor in determining its efficacy [1,2]. Existing research on drugs for polymorphism have focused on polymorphs, solvates, cocrystals, and salt formulation. In the last two decades, pharmaceutical cocrystals have been widely used in academia and industries to optimize the physicochemical properties of a given active pharmaceutical ingredient, such as stability, solubility, dissolution rate, bioavailability, and tabletability, etc. [3,4,5,6,7,8]. Hence, numerous studies on the fundamental aspects and applications of cocrystallization have been published, and several cocrystals are currently on the market or under clinical trial phases, e.g., sacubitril-disodium valsartan-water (Entresto^TM^), escitalopram oxalate-oxalic acid (Lexapro^®^), ertuglifozin-L-pyroglutamic acid and tramadol-celecoxib [9,10,11,12,13]. These indicated that cocrystal formation is an effective method for improving drug’s solubility and oral bioavailability.

**Figure 1:**
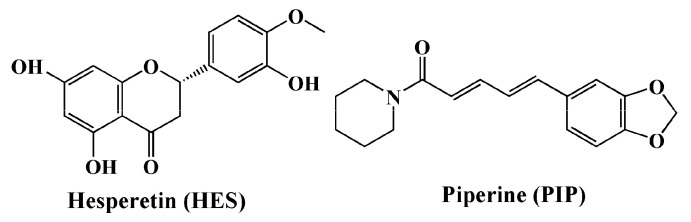
Chemical structures of HES and PIP.

HES is the aglycone of hesperidin, and is largely derived from sweet oranges and lemons belonging to the citrus species. The chemical structure of HES is shown in Figure 1 and its IUPAC name is ((S)-2,3-dihydro-5,7-dihydroxy-2-(3-hydroxy-4 methoxy-phenyl)-4H-1-benzopyran-4-one). Similar to most flavonoids, HES is also a natural antioxidant and has anti-inflammatory, anti-atherosclerotic, and anti-diabetic properties [14,15,16,17,18]. Several studies have reported that HES can ameliorates anxiety and depression-like behaviors by enhancing Glo-1 and activating the Nrf2/ARE pathway in the brain of diabetic rats and high glucose cultured SH-SY5Y cells [19]. HES is also regarded as a natural product for the prevention and treatment of cancer [20,21]. Moreover, HES and its derivatives may improve complex central nervous system diseases such as Alzheimer’s disease (AD) [19,22]. However, the clinical development of HES is limited because of poor water solubility. Numerous HES cocrystals with picolinic acid (PICO), nicotinamide (NICO), caffeine (CAFF), and temozolomide (TMZ-HSP) have been reported, the first three cocrystals in aqueous buffer showed maximum concentration of HES to be nearly four to five times higher than the pure substance, and for TMZ-HSP, the maximum solubility of HES was significantly increased by 17.8 (at pH 1.2) and 26.3 (at pH 6.8) times [23,24]. The increase in solubility of the above HES cocrystals is due to the water-soluble coforms. Currently, many other water-soluble components were selected for drugs’ cocrystals, such as nicotinamide, isonicotinamide, theobromine, theophylline, caffeine, betaine, and urea [25,26,27,28,29].

Although solubility is a major limiting factor in hydrophobic drugs’ bioavailability, the efflux of intestinal P-glycoprotein (P-gp), the metabolism of cytochrome P450, and the degradation of drugs by intestinal bacterial enzymes also significantly contribute to reduce drugs’ oral bioavailability [30,31]. Therefore, an integrated strategy that uses a suitable bioenhancer along with poorly soluble drugs to form cocrystals not only can maintain the desired supersaturation but also reduce the efflux of drugs to improve bioavailability. PIP (1-piperoylpiperidine) is an alkaloid (Figure. 1) mainly isolated from the pepper species (Piperaceae family). PIP can inhibit the functional activity of metabolic enzymes, such as CYP3A4, CYP1B1, CYP1B2, and CYP2E1; modulate drug transporters and increase drug absorption through the cell membrane by increasing the vasodilation of the gastrointestinal membrane [32,33]. The literature reported that PIP can drastically enhance the bioavailability of resveratrol (RSV) and curcumin compound when co-administrated [34,35]. In the cocrystal study of RSV and PIP, it was shown that RSV and PIP formed four cocrystals. Considering toxicity of solvent molecules, only RSV-Pip co-1 was utilized for further dissolution and pharmacokinetic experiments. Regrettably, it was found that the solubility of the RSV-Pip co-1 was lower than that of the original RSV, resulting in no improvement in the bioavailability of the cocrystal. This may be due to the fact that the solubility of PIP is low, and the molar ratio of RSV and PIP in the cocrystal is 1:2. After formation of cocrystal, the solubility of RSV is significantly reduced, leading to worse bioavailability [36]. However, for cocrystal of ursolic acid (UA) and PIP (1.5:1), the saturation solubility of UA in cocrystal combination was approximately 7-fold that of crystalline UA in an acid medium and 5.3-fold that of UA in a near neutral medium; the pharmacokinetic study of cocrystal UA in rats exhibited 5.8-fold improvement in AUC0-∞ value compared with the free solution. This enhancement in solubility of cocrystal UA-PIP arose from the hydrogen bond between the two molecules, which destroyed the long-range order of the component, and improvement of solubility helped achieve sufficient concentration in blood. Importantly, the inhibitory effect on P-gp and CYP3A4 induced by PIP, to some extent, was responsible for the prolonged systemic exposure of drugs, thereby increasing the permeability and oral efficacy of the cocrystal UA [37]. In addition, the cocrystal study of PIP and succinic acid can also prove that PIP has hydrogen bond acceptors and it can form cocrystals with molecules with hydrogen bond donor groups [38]. As a natural product, PIP had many advantages compared with other chemical entities, such as low cost due to easy availability of plant material and the extraction and isolation methods of PIP are easy and well known [39]. Most notably, it is safe to use. The current work thus selected PIP as a cocrystal former to prepare a cocrystal with HES to increase bioavailability of HES. One cocrystal of HES and PIP was obtained via multiple methods and characterized comprehensively. The crystal structure was successfully determined by single-crystal X-ray diffraction (SCXRD). The cocrystal’s solubility and bioavailability were also examined to evaluate its pharmaceutical applicability.

## 2. Materials and Methods

### 2.1. Materials

HES (purity ≥ 97%) was obtained from Shanghai Aladdin Bio-Chem Technology Co., Ltd. (Shanghai, China). PIP (purity ≥ 98%) was purchased from Shanghai Yuanye Bio-Technology Co., Ltd. (Shanghai, China). Other reagents were procured from Sigma (Shanghai, China). Deionized water was prepared using the Hitech-K flow water purification system from Hitech Instruments Co., Ltd. (Shanghai, China).

### 2.2. Methods

#### 2.2.1. Preparation of HES–PIP Cocrystal

In a typical experiment, HES and PIP at 1:1, 2:1, and 1:2 M ratios were dissolved in ethanol. The two solutions were then mixed by closing the bottle and stirring mixture. Solids were found in solution as the solubility decreased in the cocrystal. The solution was stirred continuously for 12 h at room temperature to fully crystalize. The resulting suspension was centrifuged to an isolated solid and then dried under vacuum at 50 °C for 24 h. A large amount of powder samples was achieved, with a yield of approximately 80%.

#### 2.2.2. Single Crystal X-ray Diffraction (SCXRD)

The HES–PIP cocrystal needed to be sufficiently large and robust to be analyzed by SCXRD. The supernatant remained after the powder sample prepared was collected, covered by a parafilm with several small holes, and slowly evaporated at room temperature. Block single HES–PIP cocrystals were obtained after three days. This process was also used to the comprehensively convert the reactants into the desired products without waste.

The SCXRD data on the HES-PIP cocrystal were obtained using a Bruker Smart Apex II CCD diffractometer with Mo–Kα radiation (λ = 0.71073 Å) at 296 K. The structure was solved by direct methods and refined against F^2^ using SHELXL-97 package [40,41]. All calculations were performed using SHELXTL Ver. 6.10. All figures were drawn using Mercury Ver. 3.3 [42]. The final positional and thermal parameters for the HES–PIP cocrystal are listed in the deposited CIF file, and the CCDD number is 2122688.

#### 2.2.3. Differential Scanning Calorimetry (DSC) and Thermogravimetric (TG)

DSC/DTA-TG STA 449 F5 Jupiter^®^ (NETZCH, Selb, Germany) instrument was used to test the melting point and analyze thermal behaviors of samples. The difference in the material’s crystal structure caused the change in melting point. Approximately 4–6 mg of samples (i.e., HES, PIP, or cocrystal) were placed in an aluminum pan, covered with a lid, and heated from 40 °C to 500 °C at a rate of 10 °C/min, using N_2_ as purge gas and protect gas at a flow rate of 50 mL/min. The signals of DSC and TG were collected simultaneously.

#### 2.2.4. High Performance Liquid Chromatography (HPLC)

The cocrystal’s content was determined by HPLC using Waters Delta 600 pump and a 2487 UV detector. An amount of 5 mg of cocrystal was accurately weighed and dissolved in methanol. Then 10 uL was taken for detection by HPLC. The mobile phase was a mixture of acetonitrile and water (v:v, 1:1) with a flow rate of 1 mL/min at 37 °C, and the chromatographic column was a DIKMA Diamonsil C18 reverse-phase column (250 mm × 4.6 mm, 5 μm, China). The detection wavelengths were 280 and 343 nm for HES and PIP, respectively.

#### 2.2.5. Powder X-Ray Diffraction (PXRD)

The PXRD measurements of HES, PIP, and the HES–PIP cocrystal were performed using an XRD-6100 powder X-ray diffractometer (Shimadzu Corp., Tokyo, Japan) with Cu-Kα radiation at 30 mA and 40 kV. The XRD patterns of all samples were collected from 2θ° = 4° − 60° with a scan speed of 4°/min at room temperature. The experimental PXRD result was compared with the PXRD patterns calculated from the single-crystal test to confirm the composition of cocrystal.

#### 2.2.6. Fourier Transform Infrared (FT-IR)

FT-IR studies were performed on IRAffinity-1 (SHIMADZU, Kawasaki, Japan) in the range of 4000–500 cm^−1^ (4 cm^−1^ resolution, 32 scans) via KBr pellet method. The crystal samples were weighed and ground with KBr at a certain ratio (1:100, *w*/*w*) and then compressed into tablets for analysis. The scanning frequency was 32 times, and the resolution was 4 cm^−1^.

#### 2.2.7. Solubility Experiments

The solubility of the HES–PIP cocrystal in simulated gastrointestinal fluid was measured in the present study following the method described in the literature but slightly modified [24]. The samples were previously milled and sieved (75–150 μm) before examination to reduce the effect of the crystal’s size on its solubility behavior. A specific amount (containing 25 mg of HES) of the HES–PIP cocrystal was weighed and added into a centrifuge tube containing 5 mL dissolving medium, and the suspension was incubated at 37 ± 0.2 °C rotating at 100 rpm. Taking a tube at 0.25 h, 0.5 h, 1 h, 2 h, 4 h, 6 h, 8 h, 12 h, 24 h, and 48 h, the samples were centrifuged at 12,000 rpm for 5 min, and the supernatant was diluted with methanol and analyzed by HPLC. The cocrystal’s solubility at different times was compared with that of raw HES. The solubility experiment was conducted in triplicate. The residual solid in each tube was also analyzed by PXRD. 

The cocrystal’s supersaturation factor (SF) was calculated through the drug supersaturation–time curve following the method presented in a previous study [43], and the calculation formula is as follows:

(1) $$\mathrm{SF}=\frac{{\mathrm{AUC}}_{0.25-48\;h\left(\mathrm{Cocrystal}\right)}}{{\mathrm{AUC}}_{0.25-48\;h\left(\mathrm{Hesperetin}\right)}}$$

where SF is the supersaturation factor, AUC_0.25–48 h (Cocrystal)_ is the area under the curve of the cocrystal supersaturation–time profile, and AUC_0.25–48 h (Hesperetin)_ is the area under the curve for a saturated solution.

#### 2.2.8. Bioavailability

Eighteen Sprague–Dawley rats weighing 220–250 g were randomly divided into three groups (*n* = 6), fasted for 12 h but freely given water before the experiment. The HES, the physical mixture of HES and PIP, and the HES–PIP cocrystal were delivered by gavage at a dose equivalent to 80 mg of the HES/kg body weight of the animal as a suspension in water. Next, 500 µL blood was withdrawn and placed in a centrifuge tube with heparin from the eye sockets of rats at 0.25 h, 0.5 h, 1 h, 2 h, 4 h, 6 h, 8 h, 12 h, 24 h, and 48 h after oral administration. The plasma was separated by centrifugation (4000 rpm, 15 min) and saved at −80 °C until analysis. Next, 100 µL of plasma sample was added into 400 μL of methanol and vortexed for 5 min to fully precipitate the protein [44], then centrifuged at 10,000 rpm for 10 min. The supernatant was then transferred to another tube and evaporated to remove the solvent under a stream of nitrogen. The residual solid was then dissolved in methanol with vortex oscillation for 3 min, and the mixture was centrifuged at 10,000 rpm for 10 min. Finally, the supernatant was analyzed via HPLC method for HES content. PK parameters were obtained on the basis of a model-independent method using DAS 2 program.

## 3. Results and Discussion

### 3.1. Cocrystal Screening by DSC

**Figure 2:**
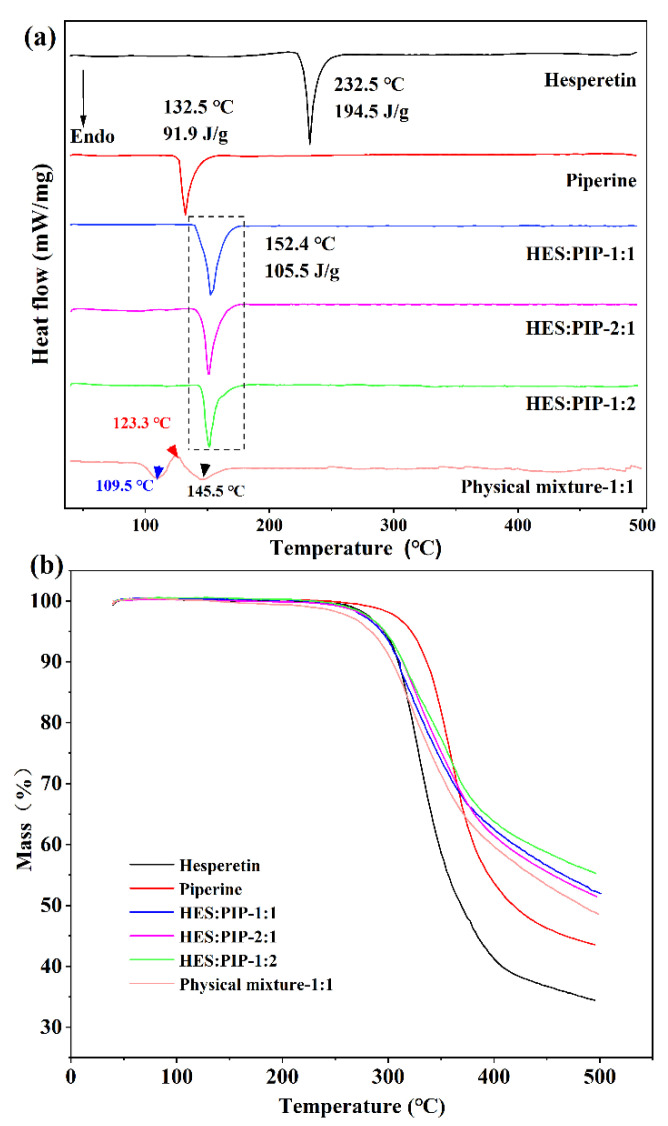
DSC (**a**) and TG (**b**) curves of HES–PIP system.

DSC and TG analysis are conventional and useful techniques to determine the thermal behaviors of solid samples. Being the simplest and fastest method for selecting systems that produce cocrystals, the DSC method was thus applied for preliminary screening [45]. In the thermogram (Figure 2a), the melting points of HES and PIP were 232.5 °C and 132.5 °C, respectively. Results revealed that when a physical mixture of HES and PIP at a molar ratio of 1:1 is heated via DSC, two endothermic peaks at 109.5 °C and 145.5 °C and a small exothermic peak at 123.3 °C can be observed. Considering the relationship between a physical mixture’s thermal behavior and cocrystal formation, an exothermic peak associated with cocrystal formation was detected immediately after the occurrence of an endothermic peak when the physical mixture consisting of two components capable of cocrystal formation was heated via DSC [46,47]. The current work thus predicted that HES and PIP will form a cocrystal. This study obtained bulk crystals in the mixed solution of HES and PIP and identified them through DSC. The thermal behavior of all crystals exhibited a single endothermic peak at 152 °C under the three molar ratios, which differ from those of the raw materials (Figure 2a). HLPC was performed to identify the crystal’s composition to rule out the possible interference of recrystallization for individual components. The result showed that the crystal contains HES and PIP, and the molar ratio of HES and PIP is 1:1 after calculation. The cocrystal system of HES–PIP was established successfully on the basis of the above results [48,49]. The TG image showed that HES, PIP, and the HES–PIP cocrystal were free from crystalline water or solvents in the lattice and begin to decompose at approximately 252.5 °C, 267.4 °C, and 254.9 °C, respectively.

### 3.2. Crystal Structure Analysis

**Figure 3:**
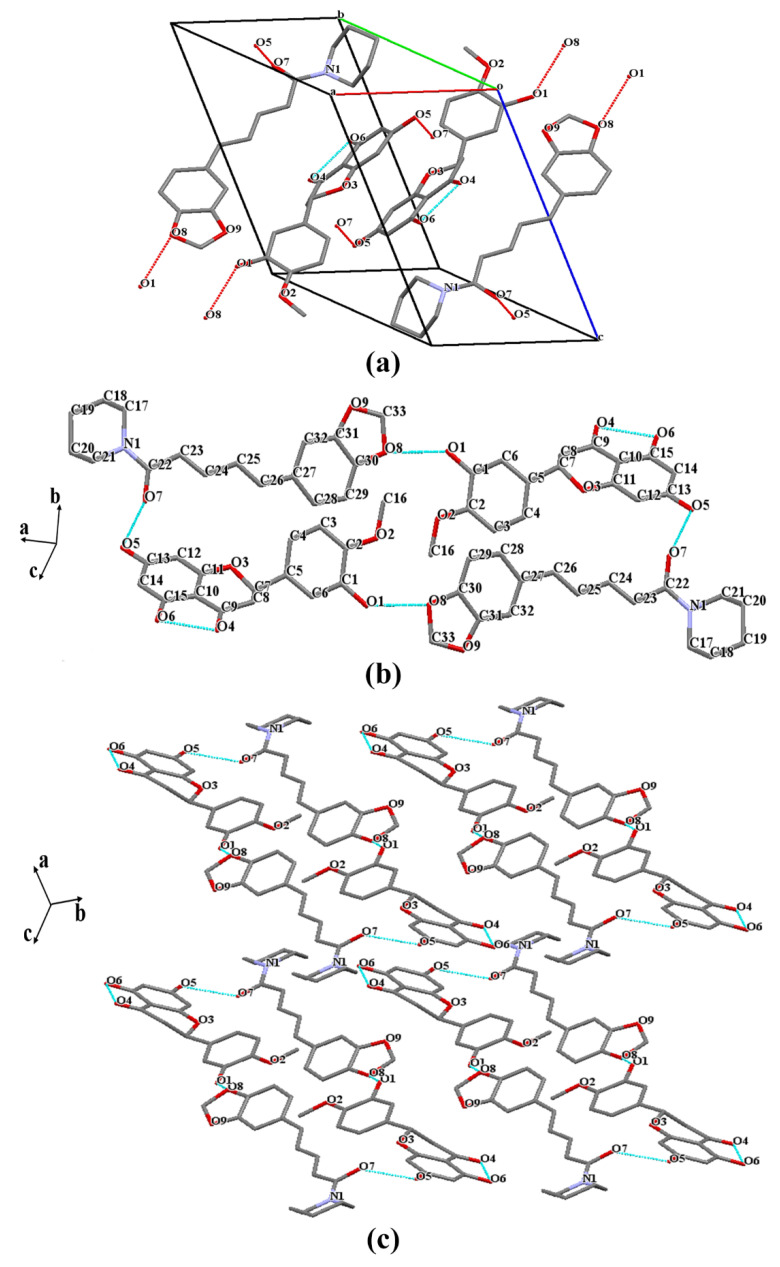
(**a**) Cell packing, (**b**) 2D and (**c**) 3D hydrogen-bonded frameworks of HES–PIP. H-bonds are represented by dashed lines.

The cocrystals’ structure including supramolecular synthons, crystal-packing details, and the location of hydrogen bonds in the supramolecular synthons can be obtained by vSCXRD analysis [23]. The crystallographic data and refinement details are depicted in Table 1. Fuji et al. [50] reported that the structure of HES is crystallized in the monoclinic space group P21/c with the following unit cell parameters: a = 12.464 (2) Å, b = 16.226 (3) Å, c = 7.102 (1) Å, α = 90°, β = 104.24 (2)°, and γ = 90°. Compared with the crystal structure of HES, the SCXRD analysis of the HES–PIP cocrystal (Figure 3a) revealed that it crystallizes in the *P-1* space group of the triclinic system which consists of one molecule of HES and one molecule of PIP in the asymmetric unit (Z = 2). As shown in Table 1, the cell length and cell angle of HES–PIP were a = 10.531 (2) Å, b = 11.879(3) Å, c = 13.363 (1) Å, and α = 105.644 (2)°, β = 111.934 (2)°, and γ = 100.486 (2)°. The information about the cocrystal is provided in Table 2 and Figure 3a. The crystal cell has two HES molecules and two PIP molecules. These molecules contact each other by O–H···O hydrogen bond interaction (O_1_H_1_···O_8_ 2.243 Å, O_5_H_5_···O_7_ 1.865 Å). This kind of connection forms a head-to-tail combination, creating a ring-shaped 2D structure (Figure 3b). The sheets are then further stacked along the C axis to form a 3D framework (Figure 3c) through the moderate π-π interaction stemming from staggered PIP molecules in two adjacent layers.

A previous study has shown that intramolecular hydrogen bonds exist at O_6_H_6A_O_4_ in the HES’s structure, and an intermolecular hydrogen bond exists between -O_5_H_5_ of one molecule and -O_6_H_6A_ of the other. However, a new connection (O_1_H_1_O_2_ 2.220 Å) appeared in the HES–PIP structure. These changes on the crystal structure indicated that HES’s physicochemical properties will be affected due to the formation changes in the of HES–PIP cocrystal’s internal hydrogen bond existence.

### 3.3. Powder PXRD Analysis

**Figure 4:**
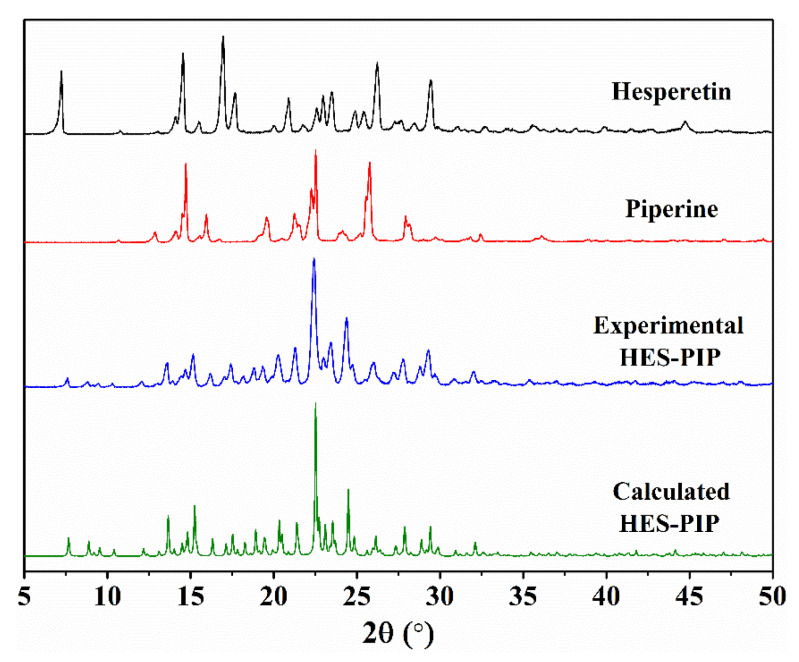
Comparison of experimental and calculated PXRD patterns of HES–PIP cocrystal, HES, PIP.

In this part, the powder sample’s PXRD pattern is compared with the simulated PXRD obtained from the SCXRD analysis, which will also prove whether the material has starting materials or impurities. As shown in Figure 4, the HES–PIP cocrystal (blue line) has diffraction peaks at the 7.65°, 8.82°, 9.52°, 10.32°, 12.12°, 13.62°, 22.42°, 23.44°, and 24.38° positions (2θ) which are absent in HES and PIP. HES exhibits characteristic reflections at 7.24°, 16.90°, 17.64°, 26.18°, and 29.42°. The characteristic peaks of PIP are at 14.70°, 15.96°, 19.54°, 22.52°, 25.81°, and 27.90°. The experimental PXRD pattern is consistent with the simulated one calculated from SCXRD data (green line), confirming the crystalline-phase purity of the achieved powder cocrystal. 

### 3.4. FTIR Analysis

**Figure 5:**
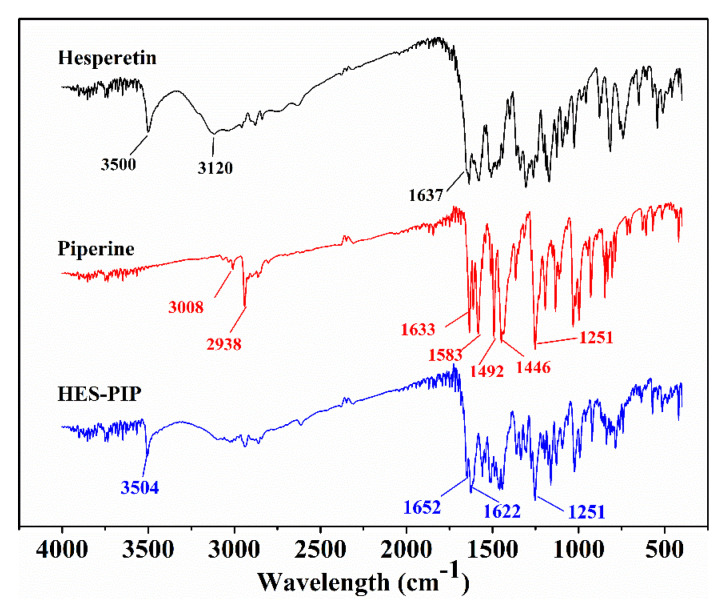
FTIR spectra of HES–PIP cocrystal, HES and PIP.

Intermolecular interactions in the HES–PIP cocrystal were completely revealed by the solved crystal structures. This study also attempted to obtain more information about the intermolecular interactions of functional groups involved in the changes in their vibrational frequencies via FTIR spectra analysis (Figure 5). For HES, the characteristic peaks appear at 3500 cm^−1^ and 1637 cm^−1^, corresponding to the O–H and C=O stretching vibrations, respectively [51]. PIP’s structure contains several functional groups, such as a benzene ring, C–O, and C–N, forming a long conjugate system with C=C bonds. The characteristic peaks located at 3008 cm^−1^, 1583 cm^−1^, 1492 cm^−1^, and 1446 cm^−1^ were attributed in the stretching vibration of -CH on the aromatic ring. The C=C and C=O stretching vibrations were observed at 2938 and 1633/1583 cm^−1^, respectively. For the HES–PIP cocrystal, the C=O stretching vibrations were observed at 1652 and 1622 cm^−1^, and the –OH stretching was shifted to 3504 cm^−1^. These red-shifts occurring in the FTIR spectra reflect the hydrogen-bonding modes accompanying cocrystal formation.

### 3.5. Analysis of the Solubility Analysis

**Figure 6:**
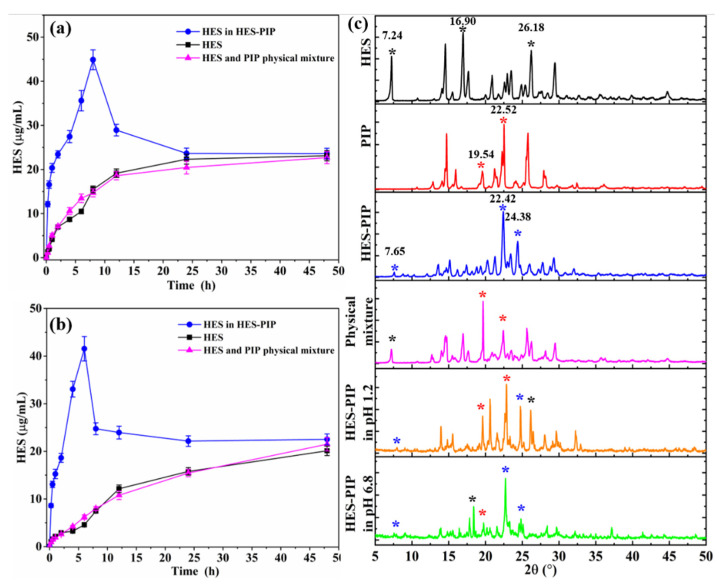
Equilibrium solubility of HES, HES in HES–PIP in simulated gastrointestinal juice (**a**, pH = 1.2), (**b**, pH = 6.8), and PXRD patterns after 48 h solubility test (**c**). The same color of symbol (*) represents the characteristic peak of the same substance.

Considering the HES’s poor water solubility, this study attempted to modify its physicochemical properties using cocrystals to improve its hydrophilicity. The result of the equilibrium solubility test on the HES–PIP cocrystal in simulated gastrointestinal fluid at different times are illustrated in Figure 6a,b. The solubility of HES in two buffers increased with time, and the dissolution rate slowed down and reached equilibrium after 12 h. Pure HES has a maximum solubility of 23.13 μg/mL (pH 1.2) and 21.12 μg/mL (pH 6.8). Moreover, the physical mixture of the two compounds could not increase HES’s solubility (pink line in Figure 6a,b). After the formation of the HES–PIP cocrystal, the solubility of HES was effectively increased to 44.89 at 8 h (pH 1.2) and 41.55 μg/mL at 6 h (pH 6.8). HES’s concentration then started dropping but remained steadily close to the levels exhibited by pure HES. The reason for this dissolution behavior was the breakdown of cocrystals to their starting molecules on extended exposure to aqueous medium, which will be further confirmed by the PXRD analysis of the residue. This peculiar effect known as the parachute effect offers a comfortable period window considered sufficient for the cocrystal to be absorbed into the systemic circulation before it releases the active constituent [52]. The degree of supersaturation as a function of time was calculated to further investigate the dissolution behavior of the HES–PIP cocrystal in vitro. The SF was expressed as the ratio of the area under the degree of HES–PIP supersaturation–time profiles up to 48 h (AUC_0.25–48 h_). The AUC_0.25–48 h_ for a HES-saturated solution was calculated to be 1.35 and 1.69 in simulated gastrointestinal fluid.

The crystalline phases of the remaining materials were also examined after equilibrium solubility experiments (48 h). The result of PXRD is shown in Figure 6c. The remaining solids mainly showed several characteristic peaks of the HES–PIP cocrystal, such as 7.65° and 24.38°, but the characteristic peaks of raw HES and PIP were also present, they are 16.90°, 26.18°, and 19.54°, 25.52°, respectively. This result implies that the HES–PIP molecules decomposed into the original molecules in the dissolution media, and will recrystallize due to poor solubility. This result also explains the change of HES’s dissolution behavior in the HES–PIP cocrystal.

### 3.6. Bioavailability Analysis 

Katherine’s “melting point-based absorption potential” model describes an interesting and potentially useful relationship between the fraction absorbed and a drug’s melting point. Generally, low-melting compounds are more likely to be well absorbed than high-melting compounds. For every 100 °C increase in melting point, the maximum dose increases by 10 times, which will provide at least 50% absorption [53]. In the current work, the melting point of the HES–PIP cocrystal was lower than that of the raw HES, which may provide better absorption in vivo. This result is confirmed by the bioavailability analysis.

**Figure 7:**
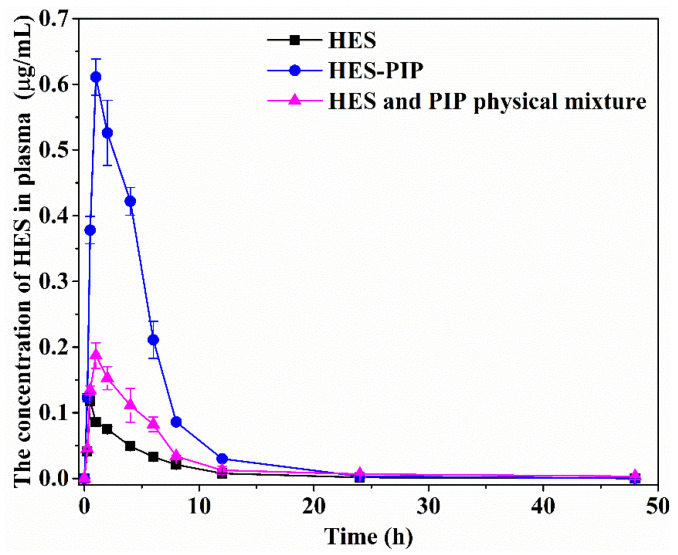
Pharmacokinetic profile of HES, HES–PIP, and the physical mixture of HES and PIP.

After oral administration and subjection of the samples to a series of tests, the plasma drug concentration–time curves of the pure HES, the HES–PIP cocrystal, and the physical mixture of HES and PIP were plotted (Figure. 7). The C_max_ and AUC_(0–t)_ of HES were 0.12 μg/mL and 0.53 µg/mL·h, while the C_max_ and AUC_(0–t)_ of the physical mixture were 0.19 μg/mL and 1.17 µg/mL·h, respectively. These results indicate that HES’s oral bioavailability can be drastically improved when co-administrated with PIP. This result is consistent with reports that PIP is a bioenhancer, that is its benefit can help in improving the absorption of insoluble drugs in vivo [54]. As shown in Figure 7 and Table 3, the C_max_ and AUC_(0–t)_ of HES–PIP cocrystal were 0.61 μg/mL and 3.23 µg/mL·h. The bioavailability of HES in HES–PIP is significantly higher than that of pure HES by six times. Moreover, the related PK parameters of pure HES, the HES–PIP cocrystal, and the physical mixture were calculated, and the results are presented in Table 3. The t_1/2_ of free pure HES, the HES–PIP cocrystal, and the physical mixture were 3.01 h, 2.68 h, and 3.26 h, while their MRT_(0–t)_ were 5.86 h, 4.47 h, and 7.86 h, respectively. Compared with pure HES, the higher plasma concentration and bioavailability of HES may be due to the better solubility of the HES–PIP cocrystal, which allows intestinal cells to easily absorb drugs. Meanwhile, PIP can inhibit the efflux of P-glycoprotein on intestinal cells to extend the retention time of a drug in vivo, which is conducive to the absorption of HES. HES cocrystals with picolinic acid, nicotinamide, and caffeine have been reported in previous research [24], and their maximum plasma concentrations were 0.63 μg, 1.15 μg, and 1.27 μg/mL, respectively. The relative bioavailability achieved was nearly 1.6 times for HESP–CAFF and HESP–NICO, 1.36 times for HESP–PICO as compared with that of pure HES, but six times for the HES–PIP cocrystal. Although solubility is lower than these three cocrystals, the HES–PIP cocrystal evidently showed a great advantage in terms of bioavailability due to the presence of PIP as bioenhancer. Therefore, the HES–PIP cocrystal is also expected to be developed into a new HES solid formulation in the future.

## 4. Conclusions

Following Etter’s rule of best donor–best acceptor pairing of hydrogen bonds, this study prepared HES cocrystals by specifically selecting coformers containing proper functional groups and biological activity. HES–PIP cocrystals were obtained, and their single-crystal structures were analyzed. These cocrystals are connected by hydrogen bonds between HES and PIP with a 1:1 stoichiometric ratio. In addition, the routine physical and chemical properties of the cocrystal were systematically characterized, and the cocrystal’s solubility and bioavailability were evaluated. As expected, the solubility and plasma concentration of the HES–PIP cocrystal significantly increased in comparison with those of pure HES. The formation of the HES–PIP cocrystal also reduced the difference of the dissolution rates between HES and PIP. These results not only provide an alternative formulation for HES but also encourage further cocrystallization trials of PIP with more compounds to be developed as an efficient oral formulation of a drug combination. These cocrystallization trials of PIP will help in overcoming the weaknesses of each parent drug.

## Figures and Tables

**Table 1:** Crystallographic Data and Structure Refinement Parameters for the HES–PIP Cocrystals.

Compound	HSP–PIP
Chemical formula	C_33_H_33_NO_9_
Formula weight	587.60
Crystal size (mm)	0.14 × 0.12 × 0.10
Temperature (K)	296 (2)
Radiation (Å)	0.71073
Crystal system	Triclinic
Space group	P −1
a (Å)	10.531 (2)
b (Å)	11.879 (3)
c (Å)	13.363 (3)
α (°)	105.644 (2)
β (°)	111.934 (2)
γ (°)	100.486 (2)
V (Å3)	1416.4 (5)
Z	2
ρ(calc) (g/cm^3^)	1.378
F (000)	620
absorp.coeff. (mm^−1^)	0.101
θ range (deg)	2.86 to 25.02
reflns collected	(R_int_ = 0.0143)
indep. reflns	4983
Refns obs. [I > 2σ(I)]	4331
data/restr/paras	4983/0/392
GOF	1.024
R_1_/wR_2_ [I > 2σ(I)]	0.0405/0.1079
R_1_/wR_2_ (all data)	0.0462/0.1071
larg peak and hole (e/Å^3^)	0.421/−0.265

**Table 2:** Hydrogen-Bonding Distances and Angles for the HES–PIP.

Hydrogen Bond	H-A (Å)	D-A (Å)	<D-H-A (Deg)	Symmetry Code
O_1_H_1_O_2_	2.220	2.667	114.55	
O_1_H_1_O_8_	2.243	2.845	130.52	x − 2, y − 1, z − 1
O_5_H_5_O_7_	1.865	2.679	171.75	−x + 1, −y + 2, −z + 1
O_6_H_6A_O_4_	1.870	2.600	147.63	

**Table 3:** Main pharmacokinetic parameters of free HES, HES–PIP cocrystal, and HES + PIP (physical mixture) in vivo (*n* = 6).

Parameters	HES	HES–PIP	HES + PIP
C_max_ (µg/mL)	0.12	0.61	0.19
T_max_ (h)	0.5	1	1
AUC_(0–t)_ (µg/mL*h)	0.53	3.23	1.17
t_1/2_ (h)	3.01	2.68	3.26
MRT_(0–t)_ (h)	5.86	4.47	7.86

## Data Availability

Not applicable.
